# Regulation of Polyvinyl Alcohol/Sulfonated Nano-TiO_2_ Hybrid Membranes Interface Promotes Diffusion Dialysis

**DOI:** 10.3390/polym13010014

**Published:** 2020-12-23

**Authors:** Yuxia Liang, Xiaonan Huang, Lanzhong Yao, Ru Xia, Ming Cao, Qianqian Ge, Weibin Zhou, Jiasheng Qian, Jibin Miao, Bin Wu

**Affiliations:** Anhui Province Key Laboratory of Environment-Friendly Polymer Materials, School of Chemistry & Chemical Engineering, Anhui University, Hefei 230601, China; 13215692963@163.com (Y.L.); 19966505287@163.com (X.H.); ylz10001@163.com (L.Y.); xiarucn@sina.com (R.X.); cmcmycyc@163.com (M.C.); gqq@mail.ustc.edu.cn (Q.G.); zwb@ahu.edu.cn (W.Z.); qianjsh@ahu.edu.cn (J.Q.)

**Keywords:** sulfonated, nano-TiO_2_, diffusion dialysis, alkali recovery, assisted transport

## Abstract

It is important to emphasize that the adjustment of an organic–inorganic interfacial chemical environment plays an important role during the separation performance of composite materials. In this paper, a series of hybrid membranes were prepared by blending polyvinyl alcohol (PVA) solution and sulfonated nano-TiO_2_ (SNT) suspension. The effects of different interfacial chemical surroundings on ions transfer were explored by regulating the dosage content of SNT. The as-prepared membranes exhibited high thermal and mechanical stability, with initial decomposition temperatures of 220–253 °C, tensile strengths of 31.5–53.4 MPa, and elongations at break of 74.5–146.0%. The membranes possessed moderate water uptake (WR) values of 90.9–101.7% and acceptable alkali resistances (swelling degrees were 187.2–206.5% and weight losses were 10.0–20.8%). The as-prepared membranes were used for the alkali recovery of a NaOH/Na_2_WO_4_ system via the diffusion dialysis process successfully. The results showed that the dialysis coefficients of OH^−^ (U_OH_) were in a range of 0.013–0.022 m/h, and separate factors (S) were in an acceptable range of 22–33. Sulfonic groups in the interfacial regions and –OH in the PVA main chains were both deemed to play corporate roles during the transport of Na^+^ and OH^−^.

## 1. Introduction

Diffusion dialysis (DD), which is driven by concentration gradient [1], is considered to be one of the most promising methods for alkaline waste water treatment as its spontaneous nature. In comparison with conventional separation processes, such as solvent extraction, precipitation, and distillation, the DD process exhibits significant superiority, including higher efficiency, low energy consumption, low installation and operating cost, stability and easiness for operation, and the environmentally friendly nature [2,3,4]. The core component of the DD process is the membrane. Therefore, membranes with excellent ion permeability and selectivity have been attracting increasing attention [5,6].

The alkaline DD process is not used as widely as it has been reported in acid recovery [7,8], which is due to the lower ion coefficients and selectivity. Recently, different efforts have been made to improve the separation performance of ion exchange membranes. These attempts could be broadly classified into three categories: new membrane-preparation methods from monomers that contain many ion exchange groups [5,9,10,11], composite membranes, and organic–inorganic hybrid membranes [12,13,14,15]. Among the rest, preparation of organic–inorganic hybrid membranes via blending or in situ methods draws the greatest attention because of their multiple functions—unique chemical reactivity, tunability of the organic polymer matrix, as well as the excellent mechanical and thermal stabilities of the inorganic backbone [3,16,17].

In recent years, organic–inorganic composites have been investigated for DD membranes with high performance [13,18]. Various nano-sized inorganic fillers such as silica [19], titania [20], zeolites [17], and montmorillonite [15,21] have already been used for the improvement of the performance of proton exchange membranes. The building of ion transfer channels between inorganic fillers and polymer matrices is the key to improve membrane separation performance [12]. However, it remains challenging to adjust the chemical environment of an interface in a composite membrane that can regulate the transportation efficiency of ions [13,14]. In our previous report [22,23,24], it was confirmed that functional groups in organic–inorganic interfacial regions could promote transport of ions. Therefore, sulfonated Nano-TiO_2_ (SNT) and polyvinyl alcohol (PVA) were chosen as an inorganic filler and a polymer matrix to prepare hybrid membranes for alkaline DD in this research. The effects of –SO_3_^−^ from SNT and –OH from PVA on the performance of hybrid membranes were discussed preliminarily.

## 2. Materials and Methods

### 2.1. Materials

PVA was supplied by Sinopharm Chemical Regent Co., Ltd. (Shanghai, China). The average degree of polymerization was 1750 ± 50. Pre-weighed PVA was immersed in water and heated to around 100 °C and kept at 100 °C for 3 h. The homogeneous solution (5.0 wt %) was cooled to 60 °C before use.

Nano-TiO_2_ powder was purchased from Nanosabz Co. Ltd. (Tehran, Iran), with average particle sizes of 30 nm, and was heated at 160 °C for 1 h before use. Sulfuric acid (H_2_SO_4_), glutaraldehyde (GA), acetone, and pure analytical toluene were purchased from Sinopharm Chemical Regent Co. Ltd. Pure analytical 1, 3-propanesultonewith was supplied by Shanghai Kang Ta chemical Co. Ltd. (Fengxian, Shanghai). Deionized water was used throughout.

### 2.2. Surface Modification of Nano-TiO_2_

**Scheme 1:**
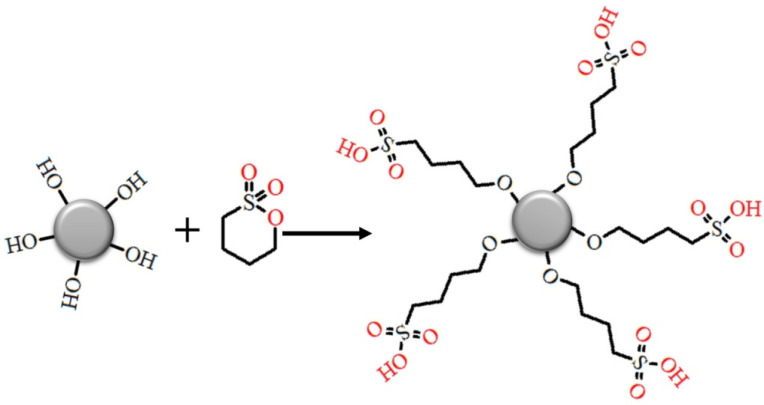
Schematic diagram of sulfonation reaction of nano-TiO_2_.

Sulfonation of nano-TiO_2_ was illustrated in previous report [17,25,26]: 1 g of pre-heated nano-TiO_2_ and 4.4 g 1, 3-propanesultone were added into 300 mL toluene under ultrasonic dispersion, and then, the mixture was stirred vigorously at 120 °C for 48 h. After that, the powders were soxhlet-extracted by acetone at 80 °C for 48 h. Finally, the samples were dried at 55 °C in a vacuum drying oven for 12 h to obtain SNT powders. The sulfonated process is presented in Scheme 1, and the sulfonation was confirmed by the FT-IR spectra, which was obtained by a NEXUS-870 (Thermo Fisher Scientific, Waltham, MA, USA) spectrometer.

### 2.3. Preparation of the Hybrid Membranes

A preweighted GA aqueous solution (with a 0.5% mass ratio) was added to a 20 mL PVA aqueous solution under stirring, and the pH value was controlled at 5. After reaction for 35 min, SNT was added into a mixture of the prepared casting solutions and PVA with different mass ratios (0%, 1%, 3%, and 5%) under high-speed shear. Then, the solutions were casted onto clean glass plates and dried at room temperature for 48 h. The obtained membranes were dried from 60 °C to 130 °C at a rate of 10 °C/h and then kept at 130 °C for 5 h. The membranes were signed as 0%, 1%, 3%, and 5%, respectively, according to the dosage of SNT.

### 2.4. Characterization and Separation Performance of the As-Prepared Membranes

The microscopic structures and basic properties of the as-prepared membranes, including FT-IR (Thermo Scientific Nicolet iS10, Waltham, MA, USA), W_R_, swelling degree, mass loss, mechanical and thermal properties (Instron 5967, Boston, MA, USA), and SEM and TEM images were measured, and the details could be seen in our previous reports [23,24]. The ion exchange capacities (IECs) of the as-prepared membranes were determined by the element analysis (Elementar Vario EL cube, Frankfurt, Germany) result of SNT. The DD test of the as-prepared membranes was detailed in our previous report [8,22], in which the effective area of membrane was 6 cm^2^ and the temperature was kept at 25 °C. The solutions were stirred during the experiment, and the membrane samples were immersed into a feed solution for 2 h before testing.

## 3. Results

### 3.1. FTIR Spectra of Original Nano-TiO_2_ and SNT and ATR-FTIR of the As-Prepared Membranes

**Figure 1:**
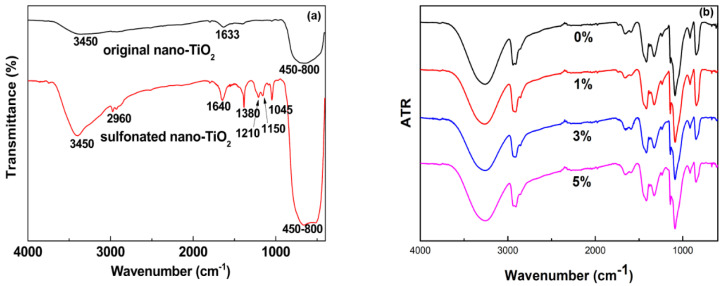
(**a**) FTIR spectra of the original nano-TiO_2_ and the sulfonated nano-TiO_2_. (**b**) ATR-FTIR spectra of the as-prepared membranes.

The FTIR spectra of the original and sulfonated nano-TiO_2_ are shown in Figure 1, in which the broad peak was observed in the region of 450–800 cm^−1^ corresponding to the Ti-O stretching vibration. The spectrum of the original nano-TiO_2_ was characterized by the broad –OH stretching vibration and the bending vibration located at 3450 cm^−1^ and 1663 cm^−1^, respectively. Compared with the original nano-TiO_2_, the presentation of the S=O symmetric vibration peak at 1150–1210 cm^−1^ and the C–H adsorption peaks at 2920 cm^−1^ and 1380 cm^−1^ confirmed the achievement of SNT [27].

The ATR-FTIR spectra of the as-prepared membranes exhibited the similar trends, except for the peak between 1000 cm^−1^ and 1100 cm^−1^, which could be assigned to C–O stretching and O–H bending vibrations coming from the PVA main chains. The detailed discussion is shown in Section 3.3.

### 3.2. IECs

The IECs of the as-prepared membranes were calculated from the results of a sulfur elemental analyzer (1.06%) and are shown in Table 1. The IEC values were in a range of 0–0.0157 mmol/g, which is much less than that of typical ion exchange membranes. This was mainly due to the lower sulfonated degree of SNT (about 1%). However, the introduction of sulfonic groups had much lower influence on the separation performance of membranes though the IECs of the as-prepared membranes than in previous reports [28,29].

### 3.3. Water Uptake (W_R_), Swelling Degree, and Mass Loss

**Figure 2:**
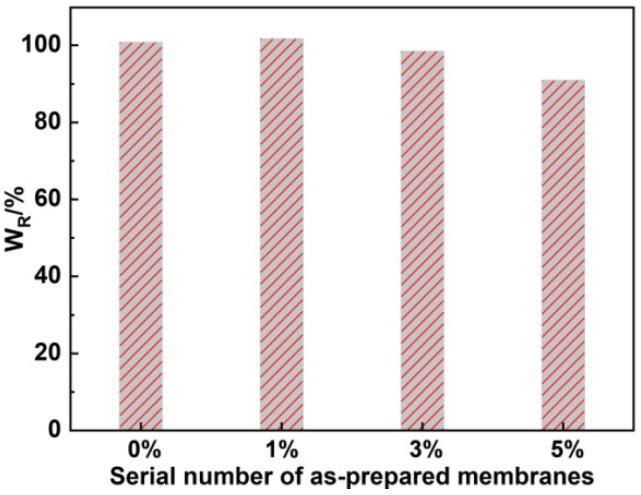
Water uptake (W_R_) of the as-prepared membranes.

**Figure 3:**
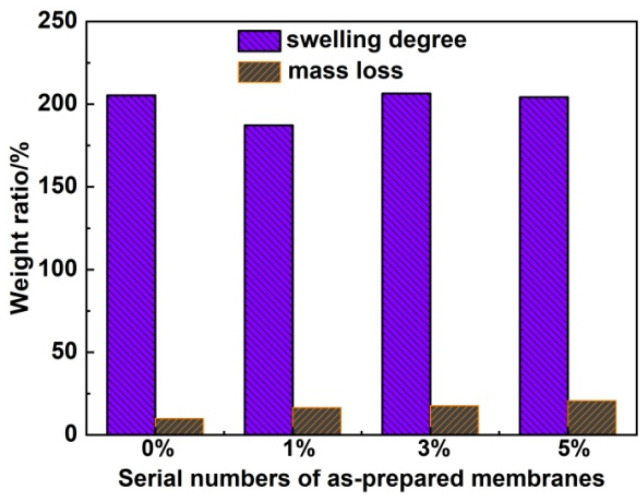
Swelling degrees and mass losses of the as-prepared membranes.

The W_R_ results are shown in Figure 2, while the swelling degrees and the mass losses of as-prepared membranes are shown in Figure 3.

The W_R_ values of the membranes were in a range of 90.9–101.7%. The effect of the loading of SNT on the W_R_ values of membranes could be explained in two aspects: The hydrophilicity of hybrid membranes increased slightly, when the dosage of SNT was 1%, which was due to the incorporation of the hydrophilic –SO_3_H and the well dispersion of the SNT particles; The values of W_R_ decreased obviously, while the dosage of SNT increased continuously. This could be attributed to the partial aggregation of the SNT, i.e., the SNT particles aggregated via the hydrogen bonds and this declined the number of dissociative –SO_3_H. Meanwhile, considering the lower sulfonated degree of SNT, the increased relative mass ratio of SNT to PVA partially led to the decreasing W_R_, when the dosage of SNT was more than 3%.

The swelling degrees of membranes in 2M NaOH at 65 °C were in a range of 187.2–206.5%, which was attributed to the degradation of crosslinked PVA chains. Membranes 1–5% exhibited slightly lower swelling degrees than that of membrane 0%, which indicated that the addition of SNT could restrict the movement of PVA chains. It could be observed obviously that the peak between 1000 cm^−^^1^ and 1100 cm^−^^1^ shifted to the higher wavenumber with the increasing dosage of SNT, which corresponded to those in a previous report [30]. The results indicated that incorporation of SNT was advantageous in the improvement of the swelling resistance of the hybrid membranes.

The mass loss of the membranes was mainly due to the dissociative PVA chains and resulted partly from the degradation of the crosslinking network under the attack of the hot alkali aqueous solution. All the membranes maintained integrity and original color after testing, while they turned brittle. This was due to the damage of the crosslinked structure caused by OH^−^. The weight losses of the as-prepared membranes were in a range of 10.0–20.8%, compared with the finding in our previous report [23], and increased with the increasing dosage of SNT. This could be attributed to the introduction of sulfonic groups to the membrane matrix, that is, free H^+^ from SNT facilitated the attack of OH^−^ and the enhanced interfacial defect aggravated the erosion of the membranes. Therefore, the mass loss of the hybrid membranes increased with the increased SNT loading increment, and thus, an appropriate dosage of SNT was necessary.

### 3.4. Mechanical Properties

The tensile strength (TS) and elongation at break (E_b_) of the as-prepared membranes are shown in Table 2. The TS values were in a range of 31.5–53.4 MPa, while the E_b_ values were in a range of 74.5–146.0%. The hybrid membranes possessed comparable mechanical strength and flexibility with those in our previous reports [22,23,31]. The strength and flexibility of the membranes declined, as the dosage content of SNT was enhanced, which indicated that the mechanical properties of the hybrid membranes were affected in an unconventional manner. Generally speaking, involvement of inorganic nanoparticles into a polymer matrix could improve mechanical properties of the composites due to the reduction of the free volume [23]. The unusual phenomena in this research could be explained as follows: –SO_3_H group in the surface of SNT was easy to interact with the –OH in PVA main chains, which might lead to the rearrangement of the PVA molecular. Meanwhile, it could improve the chance to form caves between PVA main chain and SNT because of the similar hydrophility between –OH and –SO_3_H [23]. Therefore, membranes 1%, 3%, and 5% showed declined mechanical properties compared with membrane 0%.

### 3.5. Thermal Stabilities

**Figure 4:**
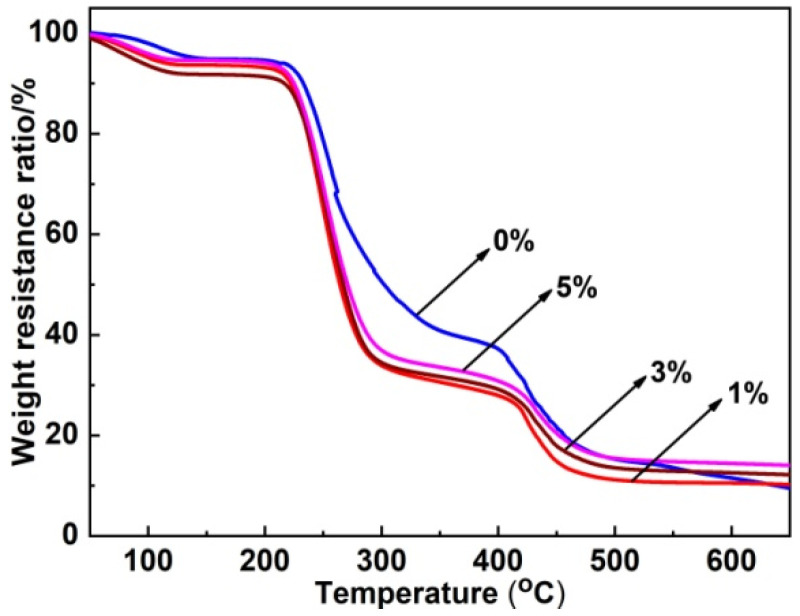
TGA results of the as-prepared membranes.

TGA testing of the as-prepared membranes is shown in Figure 4. Since the membranes were heated at 130 °C, the weight loss before 130 °C could be neglected when determining the initial decomposition temperature (IDT).

As can be see from Table 3, the IDT_1_ values were the initial decomposition temperatures determined from thermograms, which were in a range of 220–253 °C, while the IDT_2_ values were the second decomposition temperature of the other platform, which were in a range of 402–427 °C. With the increasing loading content of SNT, the IDT_1_ and IDT_2_ of hybrid membranes were higher than those of original one. The results demonstrated that introduction of SNT restricted PVA chains and enhanced the thermal stability of the hybrid membranes. The reasons were as follows: With the increasing loading content of SNT, the interaction between SNT and PVA molecules increased density of membranes and this was beneficial for the thermal stability of the membranes [27]. However, the excessive loading content of SNT could cause serious aggregation, which was disadvantage in the thermal stability of the as-prepared membranes. The crosslinking reaction was disadvantage in the dialysis of ions but advantage in the selectivity of the membranes. This would be further discussed in Section 3.7.

### 3.6. Microscopic Morphologies

**Figure 5:**
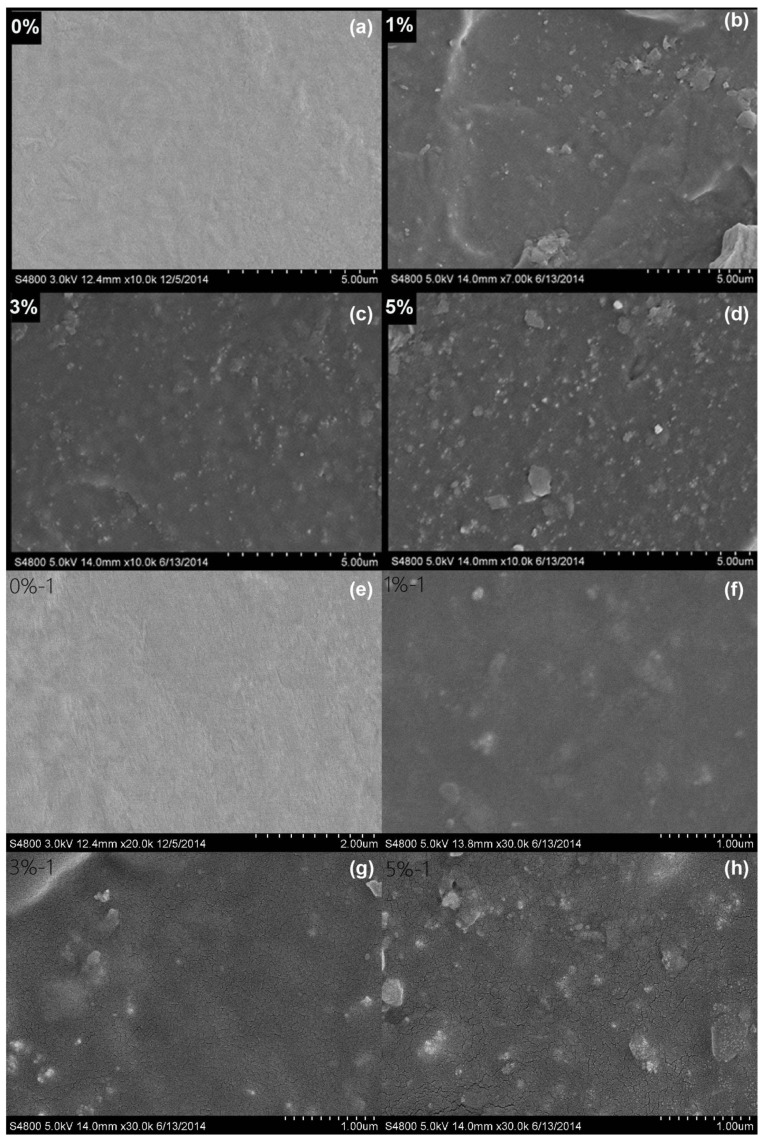
Cross-sectional SEM images of the as-prepared membranes: (**a**–**d**) 0–5% with low magnification; (**e**–**h**) 0%-1–5%-1 with high magnification.

The cross-sectional SEM pictures of the as-prepared membranes are shown in Figure 5. The hybrid membranes exhibited obvious phase interface, while the original one showed a smooth broken surface. SNT dispersed uniformly in hybrid membranes, and there were not obvious caves or structure defects in the low-magnification SEM images, even when the loading content of SNT was 5%, which indicated well compatibility between the two phases. The SNT showed some slight aggregation when the dosage content was 5%, which could be attributed to the formation of H-bonding by –SO_3_H in the surface of SNT [23]. Nonetheless, there were obvious little cracks in the high-magnification images, especially in images 3%-1 and 5%-1. This indicated that the incorporation of SNT affected the arrangement of the PVA chains and the rearrangement of the PVA chains enhanced the chance of formation of structural caves. The SEM images agreed with the analytical results of the thermal and mechanical results as well.

**Figure 6:**
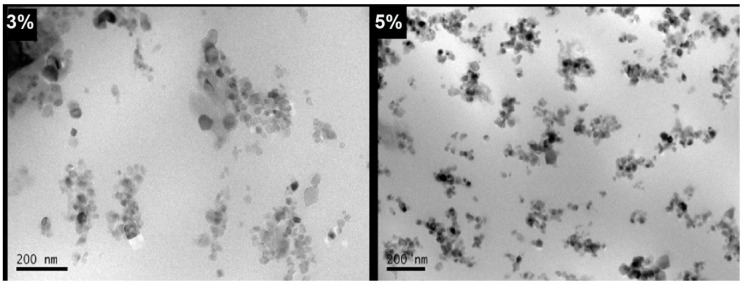
TEM photographs of membranes 3% and 5%.

To further detect the dispersion of SNT in the PVA matrix, the TEM images of samples 3% and 5% were taken, and the results are shown in Figure 6, from which we observed that SNT dispersed uniformly in membrane 3% while aggregated in membrane 5%. This agreed with the observations in the SEM images.

### 3.7. Separation Performance

#### 3.7.1. Dialysis Coefficients (U_OH_)

**Figure 7:**
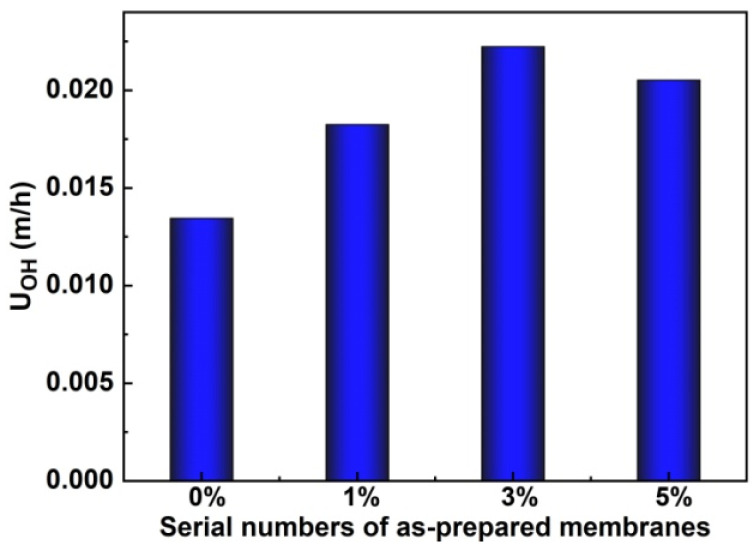
Dialysis coefficients of OH^−^ (U_OH_).

**Figure 8:**
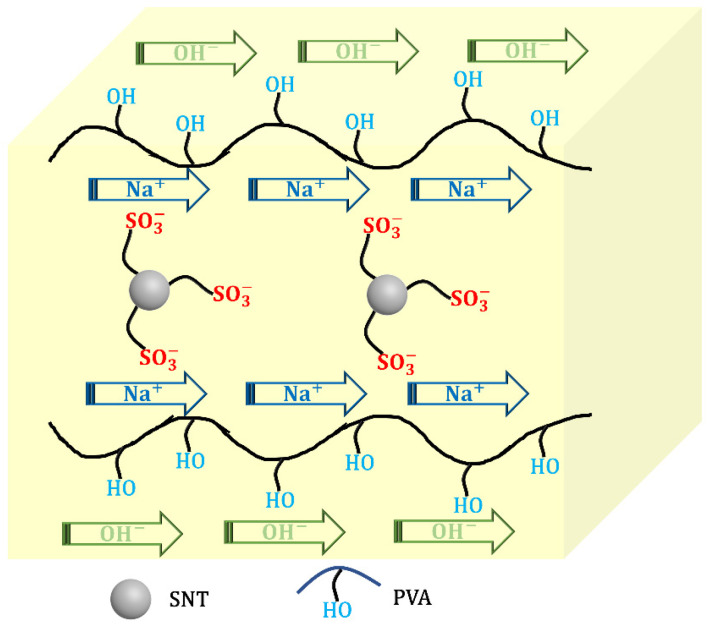
Transport schematic diagram of Na^+^ and OH^−^.

The dialysis coefficients of OH^−^ (U_OH_) are shown in Figure 7. The U_OH_ values were in a range of 0.013–0.022 m/h, which was comparable with previous reports [12,14]. As shown in Figure 7, U_OH_ values increased significantly and then decreased slightly with the increasing loading content of SNT. The results indicated that the incorporation of sulfonic groups was beneficial to the transport of OH^−^. It is known that the IEC plays an important role during ion transport because cationic ions could easily traverse through the membrane via electrostatic attraction [1]. Nonetheless, compared with typical cationic ion exchange membranes (CIEMs) [5,6], the hybrid membranes possessed two characters: IECs were far lower than those of typical CIEMs and the functional groups were in the organic–inorganic interfacial regions. Therefore, it was concluded that ion exchange groups in the organic–inorganic interfacial regions could facilitate the transport of ions. This could be explained in two aspects: On the one hand, the larger interfacial space was easier for transport of Na^+^ via electrostatic attraction; On the other hand, hydroxyl in the PVA main chains could promote transport of OH^−^ through the hydrogen bond [22]. The two factors played synergistic roles on the ion transport during the DD process and enhanced the U_OH_ to 0.022 m/h, which was nearly twice as much as the U_OH_ of a pure PVA membrane. The transport schematic diagram is shown in Figure 8. However, aggregation appeared, and membranes separation performance declined at the highest loading of 5%, which was in step with the results of SEM and TEM results. The reasons for this were as follows: –SO_3_H in the surface of SNT formed the transported channel for Na^+^ in the larger interfacial space via electrostatic attraction and this decided the separation performance of the hybrid membranes. The SNT itself aggregated via the H-bonding between –SO_3_H, and this decreased the dissociative number of –SO_3_H. Thus, the transport of Na^+^ was delayed, and the separation of membrane declined.

#### 3.7.2. Separation Factors (S)

**Figure 9:**
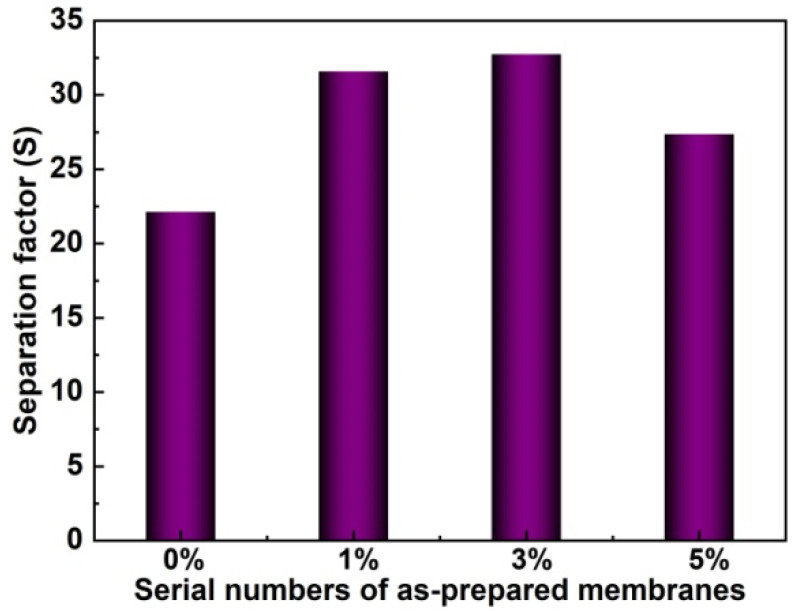
Separation factors (S) of the as-prepared membranes.

The S values of the as-prepared membranes are shown in Figure 9, from which higher S values of the hybrid membranes than that of the original one were observed. The results indicated that the incorporation of SNT was beneficial to the selectivity of membranes. The S values were in an acceptable range of 22–33, which was lower than that of SPPO-based hybrid cation exchange membranes [6,9].

The S values of the hybrid membranes increased as the loading content of SNT increased and decreased slightly while the loading content of SNT reached 5%. This could be explained as follows: All the membranes possessed high density after thermal treatment, and this would make it difficult for the transport of WO_4_^2−^ because of its bigger volume and higher valence state. On the contrary, transport of OH^−^ was less affected because of its smaller volume and lower valence state than WO_4_^2−^. More important, hydroxyl in the PVA main chains provided the assisted transport of OH^−^ via hydrogen bonding (seen in Figure 8). Meanwhile, WO_4_^2−^ suffered larger electrostatic repulsion while transport through the membrane, which was due to its multivalent property and larger volume.

Therefore, the incorporation of SNT to PVA matrix could enhance U_OH_ and S simultaneously, and this might be one of the candidates to break down the “tradeoff” effect between ion flux and selectivity. Compared with the findings in our previous report [8,22,23,24], SNT exhibited higher stability in an alkaline system than in silica, and the hybrid membranes showed excellent properties under lower dosage (less than 5%). Meanwhile, the sulfonation of nano-TiO_2_ was easy to carry out, and the blending method was easy to commercialize. However, aggregation appeared with excessive dosage of SNT, which declined the performance of membranes as discussed in Section 3.7.1. Therefore, it was important to enhance the dispersion abilities of SNT in the polymer matrix under some additional technology (such as ultrasonic dispersion, high-speed shear and in situ preparation). Thus, suitable loading content and multiple-function surface modification of SNT could help obtain membranes with the best performance. In this system, the hybrid membranes exhibited optimal performance, when the loading content of SNT was 3%, with U_OH_ and S were 0.022 m/h and 33, respectively.

## 4. Conclusions

PVA/nano-TiO_2_ hybrid membranes have been prepared by bending a precrosslinked PVA solution and an SNT suspension. The SEM and TEM images confirmed the good compatibility between these two phases. The as-prepared membranes were of good hydrophilicity and moderate alkali resistance, with W_R_ values of 90.9–101.7% and weight losses of 10.0–20.8%. The results of TGA and mechanical tests indicated that the as-prepared membranes were of thermal and mechanical stability with initial decomposition temperatures of higher than 220 °C, tensile strengths (TS) of 31.5–53.4 MPa, and elongation at break (E_b_) of 74.5–146.0%. The as-prepared membranes were applied to recover alkali from the NaOH/Na_2_WO_4_ system via the DD process successfully, and dialysis coefficients of OH^−^ (U_OH_) and separation factor (S) values were in a range of 0.013–0.022 m/h and 22–33, respectively. The sulfonic groups in the organic–inorganic interface of the hybrid membranes and –OH from PVA main chains were deemed to play important roles during the DD process: Na^+^ transported through the main channels made up of –SO_3_^−^, while OH^−^ transported through the assisted channels made up of –OH from PVA. Thus, ion flux and selectivity could enhance simultaneously by the incorporation of SNT. The membrane exhibited an optimal performance when the loading content of SNT was 3%, with U_OH_ of 0.022 m/h and S of 33.

## Figures and Tables

**Table 1:** Theoretical ion exchange capacities (IECs) and thicknesses of the as-prepared membranes.

Membranes	0%	1%	3%	5%
IEC (mmol/g)	0	0.0033	0.0096	0.0157
Thickness (μm)	68	73	76	69

**Table 2:** Tensile strength (TS) and elongation at break (E_b_) of membranes 0–5%.

Membranes	0%	1%	3%	5%
TS (MPa)	53.38	38.41	31.46	33.1
E_b_ (%)	146	108.28	90.02	74.48

**Table 3:** Initial decomposition temperatures (IDTs) of the as-prepared membranes.

Membrane	0%	1%	3%	5%
IDT_1_ (°C)	220	247	253	250
IDT_2_ (°C)	402	425	431	427

IDT_1_ was the initial decomposition temperature determined from thermograms. IDT_2_ was the second decomposition temperature of the second platform.
